# County land use carbon emission and scenario prediction in Mianyang Science and Technology City New District, Sichuan Province, China

**DOI:** 10.1038/s41598-024-60036-3

**Published:** 2024-04-23

**Authors:** Tianyi Wei, Bin Yang, Guangyu Wang, Kun Yang

**Affiliations:** 1 School of Environment and Resources, Southwest University of Science and Technology, Mianyang, 621010 Sichuan China; 2 National Remote Sensing Center, Mianyang Science and Technology City Branch, Mianyang, 621010 Sichuan China; 3 Tianfu Institute of Research and Innovation, Southwest University of Science and Technology (SWUST-TIRI), Chengdu, 610000 Sichuan China

**Keywords:** Land use, CLUE-S model, Carbon emission, Planning scenarios, Environmental sciences, Climate sciences, Atmospheric science

## Abstract

The role of carbon emissions resulting from land use change in the compilation of national greenhouse gas emission inventories is of paramount significance. This study is centered on the Mianyang Science and Technology City New Area located in Sichuan Province, China. We used the CLUE-S model and Sentinel-2A remote sensing data from 2017 to simulate and validate land use changes in 2022. Based on this validation, we established three simulation scenarios: a baseline scenario, an agricultural development scenario, and a construction development scenario. Using remote sensing data from 2022, we projected the land use for 2030. We also used CO_2_ concentration data collected in 2022 and 2023, processed the data using ArcGIS and Python, and conducted a quantitative analysis of carbon emissions under each scenario. Ultimately, the accuracy of both measured and predicted CO_2_ values for 2023 was juxtaposed and authenticated, thus concluding the investigative cycle of this study. Key findings include: (1) The accuracy of the CLUE-S model in the study area was assessed using overall accuracy, quantity disagreement and allocation disagreement indexes. In 2022, the overall accuracy is 98.19%, the quantity disagreement is 1.7%, and the allocation disagreement is 2.2%. (2) Distinct land resource utilization characteristics in scenarios, highlighting potential impacts on economic development and pollution. (3) Increased carbon emissions across scenarios, with construction development showing the highest rise (4.170%) and agricultural development the lowest (0.766%). (4) The predictive accuracy of the validation group's CO_2_ concentration values can reach 99.5%. This study proposes precise CO_2_ prediction at the county level, thus laying the groundwork for future research endeavors. Such findings are indispensable for informing carbon policy formulation and promoting low-carbon development strategies.

## Introduction

With the waning influence of the pandemic on China's production and daily life, the global economy is witnessing a surge in growth, while the global land use cover pattern undergoes profound transformations. These alterations directly influence the carbon emission profiles of various regions. Research has documented^1^ that land use change has become the second-largest contributor to greenhouse gas emissions, following fossil fuel combustion. Achieving China's “30–60” carbon peak and carbon neutrality goal is not only a prerequisite for fulfilling its climate commitments but also a crucial driver for advancing regional low-carbon development.

Land use and land management changes have been a focus point of scholarly inquiry as far back as 2003,^2^ offering updated estimations of carbon fluxes spanning from 1850 to 2000. These transformations led to the annual release of around 740 million tons of carbon into the atmosphere, significantly influencing the global carbon cycle. Contemporary research on the spatial correlation of carbon emissions stemming from land use encompasses five primary dimensions: (1) Investigation into the Relationship Between Land Use Types and Carbon Emissions: This line of inquiry delves into how distinct land use types, encompassing farmland, forests, wetlands, and urban areas, exert varied impacts on carbon emissions^3^. (2) Assessment of Land Use Change Impact on Carbon Emissions: Scholarship in this domain concentrates on evaluating the potential consequences of land use alterations (e.g., deforestation, land resettlement) on carbon emissions through simulation and observational analysis^4^. (3) Examination of Land Management Measures' Effect on Carbon Emissions: This investigation explores the influence of land management measures such as afforestation, reforestation, and land protection on carbon equilibrium, appraising their efficacy in climate change mitigation^5^. (4) Examination of Urbanization and Carbon Emissions: Investigations within this domain scrutinize the repercussions of urbanization on carbon emissions, encompassing factors such as urban sprawl, transportation emissions, and energy consumption^6^. (5) Analysis of Land Use Policies and Carbon Emissions: Scholars scrutinize the impact of various land use policies, including carbon trading, carbon tax, and ecological compensation, on carbon emissions, assessing the efficacy and sustainability of these policies^7^. This study initiates with an exploration of the impact of land use types and alterations on carbon emissions, supplemented by the evaluation of land management measures, urbanization effects, and land use policies.

Nevertheless, contemporary approaches in carbon emission research pertaining to land use exhibit certain limitations and challenges: Estimation Utilizing Remote Sensing and GIS Technology Previous research^8^ estimated carbon emissions resulting from land use and land cover changes in the Beijing–Tianjin–Hebei urban agglomeration in China using remote sensing and GIS technology. Although valuable, this approach might be constrained by its accuracy. A study proposed two monitoring, reporting, and verification (MRV) methods for national-level REDD + programs^9^. Nevertheless, deficiencies in the MRV methodology utilized in carbon emission studies concerning land use could influence the implementation and evaluation of REDD + programs. The study^10^ underscored that inconsistency in terminology could introduce uncertainty into the estimation of carbon emissions resulting from land use. Harmonizing and clearly defining relevant terms are suggested to enhance accuracy. Research^11^ identified errors in the estimation of land-use carbon emissions due to insufficient data and imprecise modeling. Global data on soil organic carbon sequestration rates were also subject to uncertainty due to scale issues and soil heterogeneity^5^. A study^12^ assessed the carbon recovery time of crop-based biofuel expansion in the tropics and found that carbon payback time may be longer than expected due to carbon emissions from crop production. A scholarly inquiry^13^ employed constraints from biomass observation data to analyze carbon emissions attributable to changes in land use and land cover from 1901 to 2012. Data acquisition and processing deficiencies were identified as potential sources of uncertainty. The study^14^ modeled the Earth system response to negative carbon emissions, highlighting challenges associated with model uncertainty and insufficient data. Research^15^ integrated global data on land use scenarios from 1500 to 2100, yet acknowledged uncertainty in estimating carbon emissions associated with land use due to inadequate historical data accessibility and modeling techniques. A study^16^ attempted to detect large-scale crop acreage and major crop types from Landsat data using advanced machine learning algorithms. Nonetheless, challenges regarding accuracy in estimating carbon emissions resulting from land use endured due to insufficient precision in remote sensing data processing and algorithms. The models, methodologies, and data employed in the investigations are plagued by issues concerning low accuracy and result uncertainty.

Presently, scholars have to some degree delved into the coupling relationship between regional land use change and carbon emissions, along with the associated influencing factors. Nonetheless, prevailing research perspectives largely hinge on static assessments of the current land use scenario within the study area. Most research has centered around larger scales such as the national level^17–19^, provinces and cities^20–22^, and watersheds^17,23^. There exists a notable dearth of research at the county level, particularly in the examination of carbon revenue and expenditure trends predicated on prognostications of forthcoming land use alterations within counties. There is also a gap in the analysis of carbon balance trends based on predictions of future land use changes at the county level. Counties serve as the fundamental units of social and economic development, and it is imperative to study land use at this scale with high precision. Differences in land use resulting from various development plans can be substantial and play a pivotal role in determining the distribution of regional resources and the overall quality of development.

In this paper, we have chosen to utilize the CLUE-S model, revered for its outstanding precision in small-scale regional inquiries and its capability to integrate numerous influential factors, to scrutinize scenarios at the county level. A previous study^24^ employed the CLUE-S model to downscale land use change scenarios from a global perspective to the European region, facilitating an evaluation of European landscape dynamics. This study attested to the commendable performance of the CLUE-S model across diverse scales. Furthermore,^25^ focused on sustainable land use development in northern Kosovo, combining normative scenarios with simulation modeling techniques. This research underscored the CLUE-S model's proficiency in exploring future land use scenarios. Additionally, the CLUE-S model was utilized to project global urban expansion until 2030, allowing for an analysis of its direct impacts on biodiversity and carbon storage^26^. This study underscored the model's advantageous spatial scaling and land use change modeling capabilities in the context of global urbanization projections. In another investigation^27^, the CLUE-S model was employed to investigate the effects of urbanization on regional carbon emissions. This research highlighted the model's capacity to consider the intricate interplay between human activities and environmental systems. Additionally, a study^28^ explored issues related to measurement variables' errors and spatial autocorrelation within pathway modeling. The study revealed that the CLUE-S model is adept at addressing spatial autocorrelation and measurement errors. Furthermore, an assessment^29^ delved into the impact of urbanization on carbon sinks through integrated models and urban–rural gradients derived from remote sensing data. This study affirmed the CLUE-S model's competence in scrutinizing the impact of urbanization on carbon emissions. Moreover, a comprehensive review^30^ summarized global land grab research and highlighted the CLUE-S model’s effectiveness in studying global land use changes. This research emphasized the model's utility in assessing the impact of land use changes on ecosystem services.

The merits of utilizing the CLUE-S model in the realm of analyzing carbon emissions stemming from land use are remarkable: (1) Multi-Scale Land Use Changes: The CLUE-S model's capacity to simulate land use changes at various spatial scales, from local to global, aids in understanding the diverse factors influencing carbon emissions across different scales^31^. (2) Integration of Anthropogenic Factors: The model can seamlessly incorporate anthropogenic factors like urbanization, agricultural expansion, and industrial development, all of which play pivotal roles in carbon emissions^32^. (3) Consideration of Land Use Policies: By accommodating the influence of land use policies on land utilization and carbon emissions, the CLUE-S model contributes to the formulation of more effective emission reduction policies^33^. (4) Predictive Power for Future Emissions: The model's ability to forecast future changes in carbon emissions under different scenarios offers valuable decision-making support for carbon emission reduction strategies and climate change adaptation^34^. (5) Detailed Spatial Distribution: Providing intricate spatial details of land use and carbon emissions allows for the identification of high-emission regions and potential areas for emission reduction. (6) High Flexibility: The model's adaptability to varying parameters based on distinct study areas and objectives enhances its applicability^35^. (7) Results Visualization: Visualization of results through maps and charts facilitates comprehension and communication of research outcomes among scholars and decision-makers. (8) Data-Driven Approach: Leveraging extensive field observation data for simulation enhances model accuracy and credibility. (9) Interdisciplinary Utility: The CLUE-S model transcends disciplinary boundaries, finding applications in diverse areas such as carbon emissions, ecosystem services, and land planning^36^. (10) Support for Sustainable Development Decision-Making: By elucidating the relationship between land use and carbon emissions, the model furnishes a scientific foundation for informed sustainable development decisions^37^.

Expanding on the capabilities of the CLUE-S model, prior scholars have consistently asserted that construction and transportation land are the predominant and substantial sources of carbon emissions. Furthermore, the regional absorption of carbon is intricately intertwined with the proportion of ecological land areas, such as woodlands and grasslands. Therefore, for the purposes of this study, the author elected to employ the CO_2_ concentration data for the study area in 2022, meticulously measured by their own research team. This methodology diverges from the conventional practice of estimating CO_2_ concentration values, as it circumvents potential sources of error, including seasonal variations, temperature fluctuations, human activities, and weather conditions. Thus, the utilization of authentic CO_2_ concentration data obtained in 2022 affords a more precise depiction of the real-world scenario, guaranteeing the reliability of the study's conclusions.

In accordance with this methodology, the current study centers its investigation on the county level, utilizing Sentinel-2A data for the Mianyang Science and Technology City New Area spanning from 2017 to 2022. A supervised classification methodology, employing the Random Forest image classification tool, was utilized to produce a land use classification map. Furthermore, an Auto-Logistic regression model was applied to select seven pivotal factors, encompassing roads, rivers, slope direction, population density, among others. These factors were integrated into the CLUE-S model, facilitating the accurate simulation and validation of land use changes in Mianyang Science and Technology City New District for the year 2022. Expanding upon this groundwork, the study advances to forecast the land use distribution pattern for the Mianyang Science and Technology City New Area in 2030. This prediction is achieved by configuring multiple land use change scenarios and accounting for land use-related carbon emissions under each scenario. This multifaceted approach serves multiple crucial objectives: (1) It facilitates a quantitative analysis and discourse on CO_2_ concentration levels within the new area of Mianyang Science and Technology City, elucidating the environmental ramifications of land use alterations. (2) The study extends the application of the CLUE-S model to a small-scale study area, demonstrating its utility in predicting carbon emissions. (3) By harnessing quantitative remote sensing methodologies and employing counties as the fundamental unit of analysis, the study attains high-precision forecasts of surface CO_2_ concentration. This innovative approach paves the way for new directions and insights in the realm of CO_2_ research.

## Data and methods

### Study area

The study area is situated within the Mianyang City (China Science and Technology City) Science and Technology New Zone in Sichuan Province. This region was officially designated as the Mianyang Science and Technology City New Zone on December 23, 2020, pursuant to the resolution of the People's Government of Sichuan Province. The study area is located between 104° 28′ and 104° 44′ E longitude and 31° 24′ and 31° 36′ N latitude. It covers an area of approximately 396 km^2^, representing a relatively compact zone undergoing rapid development. This area serves as a representative case for the examination of the relationship between land use and its impact on carbon emissions.

**Figure 1:**
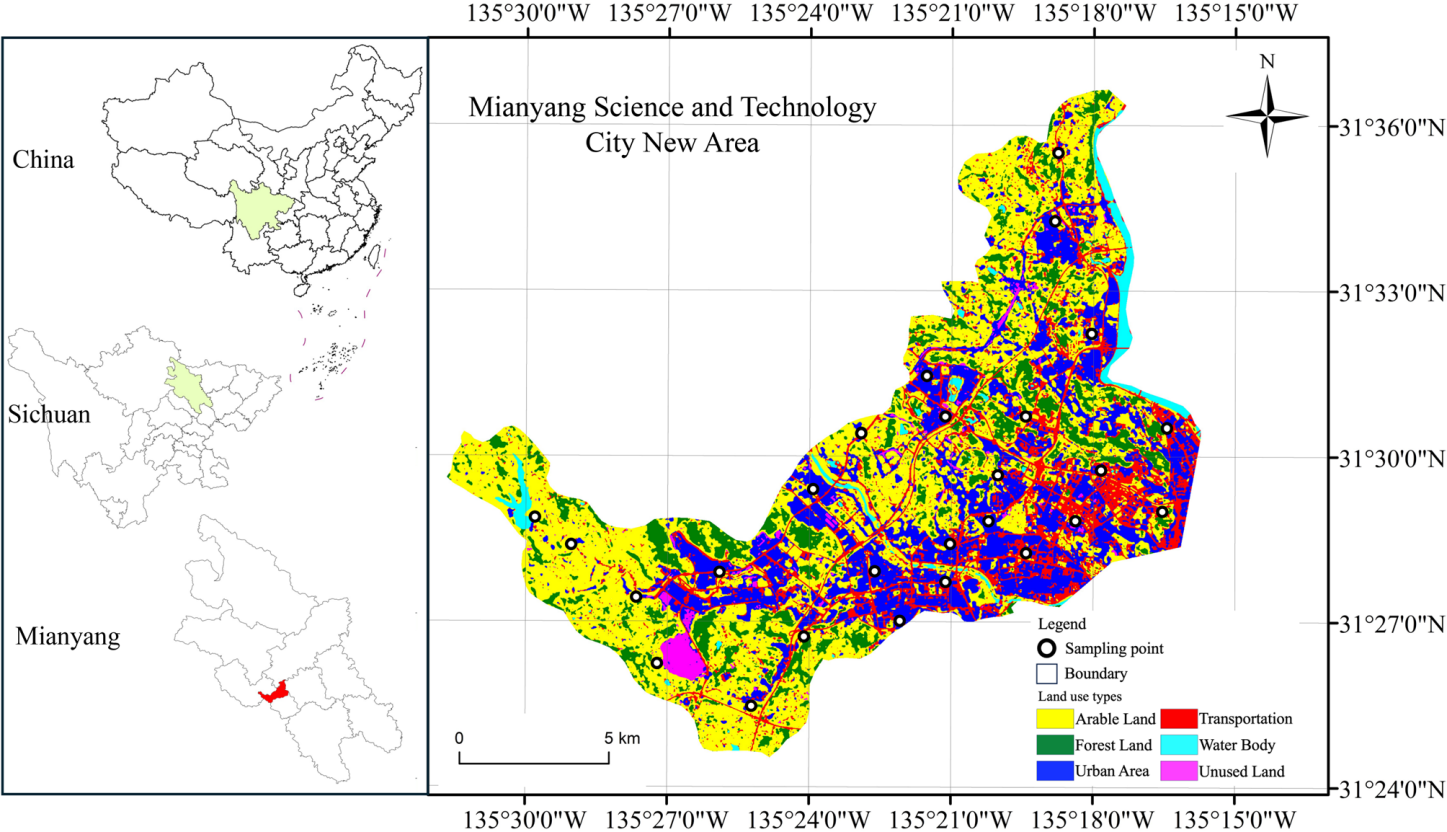
The study area for the research on County Land Use Carbon Emission and Scenario Prediction is the newly designated Science and Technology City New Area in Mianyang City, Sichuan Province, China. Land use classification maps were obtained using Sentinel-2 satellite remote sensing data through the new version of random forest supervised classification. The map includes six major land use types used in the study and the location information of 26 CO_2_ concentration sampling points. Generated in the ArcGIS 10.6 software (www.esri.com).

The study area includes the primary land categories outlined in the IPCC (Intergovernmental Panel on Climate Change) guidelines for land use classification. These categories encompass Arable Land, Forest Land, Urban Area, Transportation, Water Body, Unused Land. The diversity of land categories in the study area is advantageous for our research, and we can collect first-hand data on CO_2_ concentrations. Therefore, the study area was selected, and sampling points for CO_2_ concentration measurement were designated Fig. 1.

### Data processing flow

**Figure 2:**
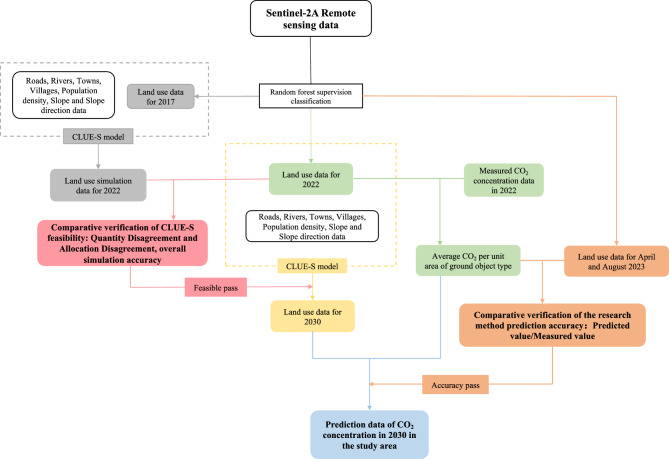
The data processing flowchart for this study includes the main line of data processing in the middle, feasibility validation steps for using the CLUE-S model in the study area on the left side, and accuracy validation steps for this study on the right side. Generated in the Microsoft Office Word 2023 (https://www.microsoft.com).

Remote sensing technology plays a pivotal role in monitoring and analyzing land use and land cover. In recent years, the rapid advancement of deep learning technology, with techniques such as convolutional neural networks (CNNs) and recurrent neural networks (RNNs), has significantly improved the field of remote sensing image analysis. Among these, the remote sensing CLUE-S model, as a rapidly evolving pre-training model, has achieved significant milestones in the domain of land use and land cover research. The research roadmap is as follows Fig. 2, and we will discuss the following topics in this research:

### Data sources and processing

#### Remote sensing data

The land use data used in this study were derived from Sentinel-2A remote sensing data. These data were acquired from the Copernicus Open Access Hub (https://scihub.copernicus.eu). The images were processed using ENVI software, including projection, correction, calibration, and resampling to a 10-m resolution, and then by the new version of the Random Forest Image Classification tool supervised classification to get the 2015–2022 land use data. After the initial classification, post-processing was performed to identify six major land categories: Arable Land, Forest Land, Urban Area, Transportation, Water Body, Unused Land. After experimenting with ten different supervised classification methods, we carefully selected the latest version of the Random Forest method that proved to be suitable for our study area. To ensure classification quality, a minimum of 100 sample points were selected for each land cover type, and multiple rounds of testing and refinement were conducted. Ultimately, we obtained eight years' worth of supervised classification results. Classification accuracy was characterized using Jeffries–Matusita (JM) and Transformed Divergence (TD) parameters. With the exception of Arable Land and Forest Land, which achieved accuracies of 1.97, and Urban Area and Transportation with an accuracy of 1.96, all other land cover categories achieved a separation accuracy of 2.00.

**Figure 3:**
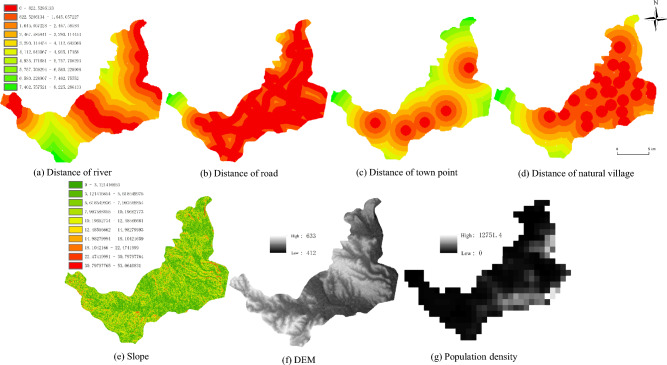
The main influencing factor data selected for the study include the Euclidean distance of extracted river (**a**), road (**b**), town point (**c**), and natural village (**d**), as well as the slope aspect (**e**), digital elevation model (**f**), and population density (**g**) influencing factors. Generated in the ArcGIS 10.6 software (www.esri.com).

In addition to remote sensing data, various data sources, including Road, River, Township, and Natural Village Factors, were integrated into this study, and subjected to specific processing steps. These factors were extracted in ArcGIS using the 2022 land use data (Fig. 3a–d). Subsequently, they were exported as raster data in ASCII format with Euclidean distance values, as required for input into the CLUE-S model. Topographic Data: Elevation data with a 30-m resolution was obtained from the Geographic Data Space and is accessible at (http://www.gscloud.cn). This dataset was employed to derive slope and slope direction data for the study area (Fig. 3e,f), which were then converted into ASCII format for further analysis. Population density data were obtained from World POP and can be accessed at (https://www.worldpop.org) as shown in Fig. 3g.

#### Carbon dioxide (CO_2_) data

To measure carbon dioxide concentrations, an unmanned aerial CO_2_ concentration monitoring instrument was utilized. The instrument is developed and produced by Chinese private enterprises and has undergone rigorous technical inspections. It is characterized by its high accuracy, capable of measuring with precision up to 1 ppm (parts per million), which equals one part of CO_2_ in a million parts of air. To capture the spatial distribution of CO_2_ concentration within the study area, measurements were conducted at 26 distinct points within the new area of Mianyang Science and Technology City.

Data collection took place from January 2022 to December 2023, with readings recorded three times per month. The instrument operated for an average of 3 min per measurement session at each measurement point. Roughly 500 data points were gathered at each measurement location. As a result, it took approximately 7 h to complete the data collection process for all 26 points. A comprehensive depiction of the data collection process in 2022 is furnished in Table 1, while data from 2023 is employed for verification purposes.

**Table 1:** The detailed information table for CO_2_ concentration sampling includes the date, weather, mean temperature, and AQI index.

Acquisition time		Weather	Mean temperature*	AQI**	Acquisition time		Weather	Mean temperature*	AQI**
Jan	05th	Fine	6.3	201	Jul	06th	Fine	32.7	39
	14th	Cloudy	6.6	195		17th	Cloudy	30.3	32
	24th	Fine	7.4	209		27th	Fine	25.6	40
Feb	07th	Cloudy	3.3	188	Aug	02th	Fine	27.9	29
	16th	Cloudy	9.9	164		19th	Fine	33.1	37
	25th	Fine	7.7	173		27th	Cloudy	28	22
Mar	10th	Cloudy	19.3	143	Sep	06th	Fine	25.3	77
	18th	Fine	22.3	122		14th	Fine	19.8	92
	27th	Fine	14.5	131		25th	Fine	18.8	63
Apr	04th	Cloudy	12.8	101	Oct	07th	Fine	15.3	104
	16th	Fine	10.6	98		16th	Fine	20.2	112
	27th	Fine	21.9	112		22th	Cloudy	21	119
May	04th	Fine	21	94	Nov	07th	Cloudy	16.5	152
	14th	Fine	15.1	82		13th	Fine	13	133
	28th	Fine	22.2	76		23th	Cloudy	13.8	162
Jun	05th	Fine	24.4	55	Dec	01th	Cloudy	4.7	189
	12th	Fine	24.4	49		12th	Cloudy	8	174
	26th	Fine	25.4	47		20th	Cloudy	7.7	191

*The unit of temperature is Celsius.

**AQI(CN) 0–50 is Excellent; 51–100 is good; 101–150 is light pollution; 151–200 is moderate pollution; 201–300 is heavy pollution; > 300 is serious pollution. Data is provided by BreezoMeter and Qweather and reflects data from air monitoring stations.

**Figure 4:**
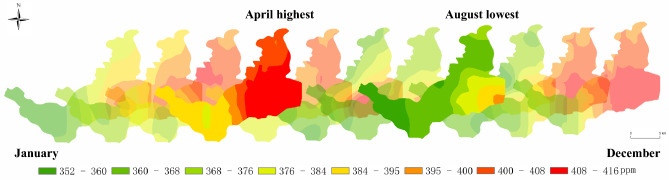
The distribution map of monthly average CO_2_ concentrations in the study area for the year 2022 (the year when unit area CO_2_ concentration values were obtained) shows the highest concentration in April and the lowest concentration in August. The results align with the conclusions of previous research papers and is positively correlated with the AQI index recorded in Table 1. It corroborates the accuracy of the study data acquisition. Generated in the ArcGIS 10.6 software (www.esri.com).

Since the data collected by the instrument consist of point measurements, it was necessary to derive the spatial distribution characteristics of overall CO_2_ concentration within the study area. To achieve this, three points were uniformly selected as validation points, while the remaining 23 points served as interpolation points. Python scripts were employed to implement and compare three different interpolation methods: kriging, inverse distance weighting, and BP (Backpropagation) neural network. The evaluation criterion for judging the effectiveness of these methods was the Root Mean Squared Error (RMSE), with smaller RMSE values indicating more effective interpolation and larger RMSE values indicating poorer interpolation performance. Table 2 displays the results of the analysis. Based on the results in Table 2, the kriging interpolation method was selected as the optimal choice due to its consistently low RMSE values across all four seasons. Subsequently, the kriging interpolation method was employed to detrend the CO_2_ data by removing the model trend^38^. Variational function analysis was performed on the residual dataset, allowing for the creation of spatial distribution maps that depict CO_2_ concentration after cross-validation^39^. Statistical analysis of the CO_2_ concentration data was performed using SPSS and Excel software. Average concentration maps for each month were generated by plotting the data using Origin and ArcGIS software, as shown in Fig. 4.

**Table 2:** The Root Mean Square Error (RMSE) obtained from three different interpolation methods, where a smaller value indicates a better interpolation effect (units: ppm).

Spatial interpolation method	Spring RMSE	Summer RMSE	Autumn RMSE	Winter RMSE
Kriging interpolation	5.494089	3.608426	0.2411935	2.335347
Inverse distance weight	6.529146	5.754797	3.520913	3.236901
BP neural network	6.490193	7.732	3.5118673	5.122628

We chose the method of monitoring at three time points each month and calculating the average primarily considering the feasibility of data collection and the aim to comprehensively capture monthly trends. This sampling frequency allows us to maintain the effectiveness and cost-efficiency of operations while obtaining relatively stable and reliable representative data for CO_2_ concentrations throughout the entire month. By monitoring at three time points within the month, we can effectively smooth short-term fluctuations and reduce data noise caused by local variations. This approach aids in furnishing more stable and representative monthly averages, thereby better reflecting overall trends rather than being influenced by isolated events or specific time points.

**Figure 5:**
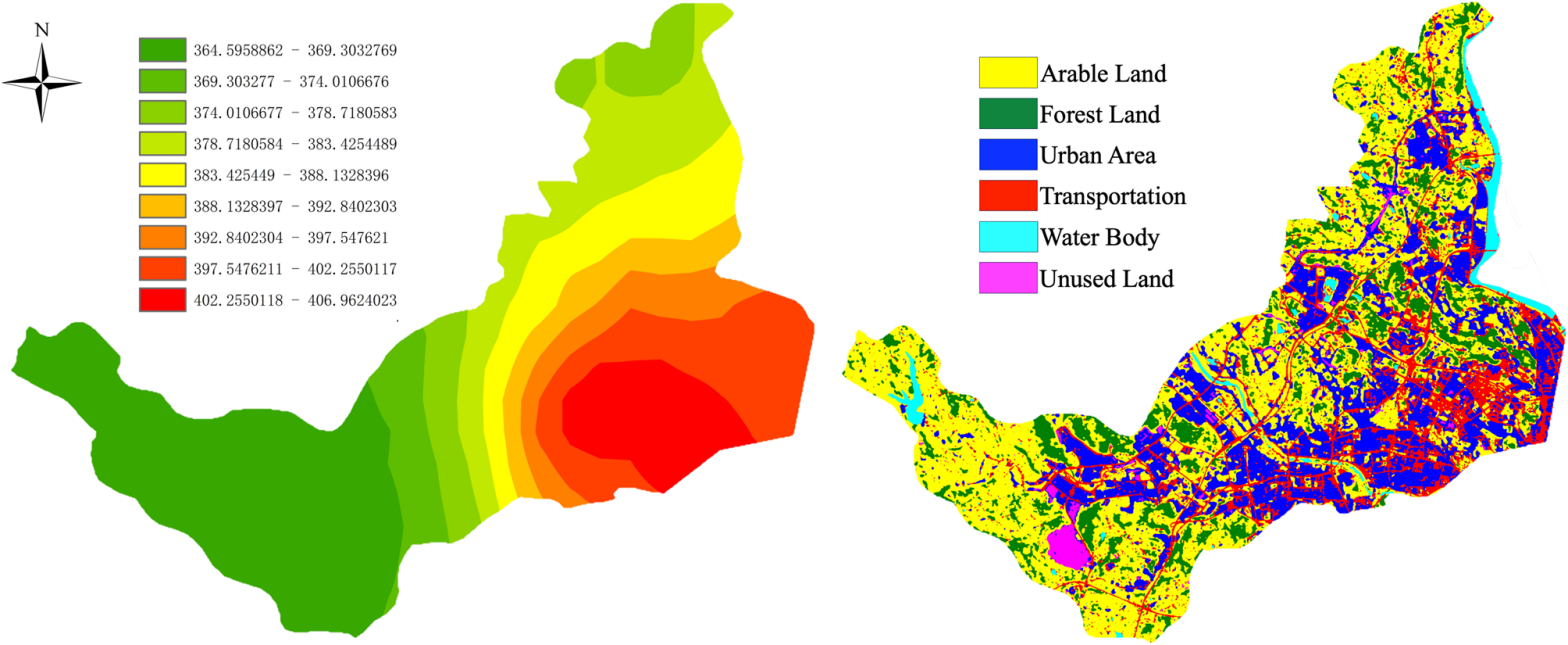
The distribution map of the annual average CO_2_ concentration for the year 2022 (left; units: ppm) and the land use classification map for the year 2022 (right). Generated in the ArcGIS 10.6 software (www.esri.com).

Following the averaging of CO_2_ data for each month, a comprehensive map depicting the distribution of average CO_2_ concentration throughout the entirety of 2022 was generated (Fig. 5, left). This map concurs with empirical observations, illustrating that CO_2_ concentration tends to be elevated in towns situated in the southeastern region and diminishes gradually towards the periphery. Additionally, this distribution map of CO_2_ concentration was juxtaposed with the 2022 land use map of the new area of Mianyang Science and Technology City (Fig. 5, right).

The main goal of this paper is to predict future trends in surface CO_2_ concentrations while controlling for various factors. To achieve this, the average CO_2_ concentration values derived from three consecutive measurements for each month were utilized. This approach effectively reduces errors related to seasonality, temperature fluctuations, human activities, and weather variations, making it more applicable to real-world conditions than the conventional method of calculating CO_2_ concentrations.

To achieve this, we used Python scripts to extract CO_2_ concentration values for each land category on a per-unit-area basis. Below is an overview of the core code utilized (The full Python code is shown in the appendix): (1) Vector Map Traversal: The code begins by traversing the two vector maps pixel by pixel, with a specific focus on filtering the white portions for faster recognition. The code stores the color of the current pixel in BGR format. (2) Color Correspondence: After processing the vector maps, the code identifies the content corresponding to each color in the image. This enables the extraction of coordinates for Arable Land, Forest Land, Urban Area, Transportation, Water Body, Unused Land from the vector map. Additionally, the coordinates of areas with median concentrations of the nine CO_2_ values are identified on the vector map. (3) The code creates a CO_2_ concentration image by generating an image of the same size as the vector map and mapping the coordinates of each land category to their respective CO_2_ concentration values. (4) Extraction and Averaging: Finally, the code extracts the CO_2_ concentration values for each land category one by one and calculates their averages. These averaged CO_2_ concentration values per unit area, which are retained to six decimal places, are then used for the calculation of CO_2_ values in 2030 and to verify CO_2_ values for 2023 (Table 3).

**Table 3:** The CO_2_ concentration values per unit area for different land use types (units: ppm).

Land classify	Arable land	Forest land	Urban area	Transportation	Water body	Unused land
Concentration	368.2222229	383.3471659	397.7473112	402.3450751	379.3919228	364.5272401

### Modeling and validation

#### Auto-logistic regression models

The CLUE-S model typically employs a binary logistic regression model to analyze the factors influencing land use change. This approach aims to reveal the causal relationship between different land use types and the factors driving changes in land use patterns within the study area. The Auto-Logistic regression model serves as a valuable extension that improves the precision of simulation results and provides a more accurate representation of the actual land use distribution patterns.

1 $$ \ln \left[ {\frac{P}{1 - P}} \right] = \alpha + \beta_{1} x_{1} + \beta_{2} x_{2} + \cdots + \beta_{n} AutoValue $$

Parameter P signifies the probability that a particular land type may occur, α is the constant of the regression equation, x_1_, x_2_, … x_n_ are the factors affecting the distribution of landforms, *β*_*1*_*, β*_*2*_*, … , β*_*n*_ are regression coefficients, Auto-Value is the spatial autocorrelation factor^40^.

#### The CLUE-S model

The remote sensing CLUE-S model, which stands for Cross-lingual Pre-training with Unified Encoding and Supervision, is a deep learning-based pre-training model. It was developed to learn comprehensive semantic information and land-use features through self-supervised learning using extensive cross-lingual remote sensing data. This model integrates the concepts of multilingual pre-training and unified encoding, enabling effective analysis of land use data across various languages and regions. The CLUE-S model is designed to specifically analyze and predict land use changes in a particular region. It achieves this by considering two categories of drivers: biophysical and socio-economic factors, as referenced in previous studies^35,41^. This model offers a clear framework and explanation for spatial and temporal changes in land use within the region. The formula is used to express its functionality.

2 $$ TPROP_{u,i} = ELAS_{i} + ITER_{i} + P_{u,i} $$

Here, TPROPu,i represents the overall suitability of raster 'u' for land use type 'i'. ELASi denotes the transfer elasticity coefficient for land type ‘i’, signifying the conversion cost of the respective land use type. ITERi stands for the competition factor of land type 'i', which is automatically adjusted during the iterative process of the model simulation. Pu,i signifies the probability distribution of raster 'u' for land use type 'i', derived through regression analysis based on the current state of land use and various driving factors.

1. Land use transfer rules

**Figure 6:**
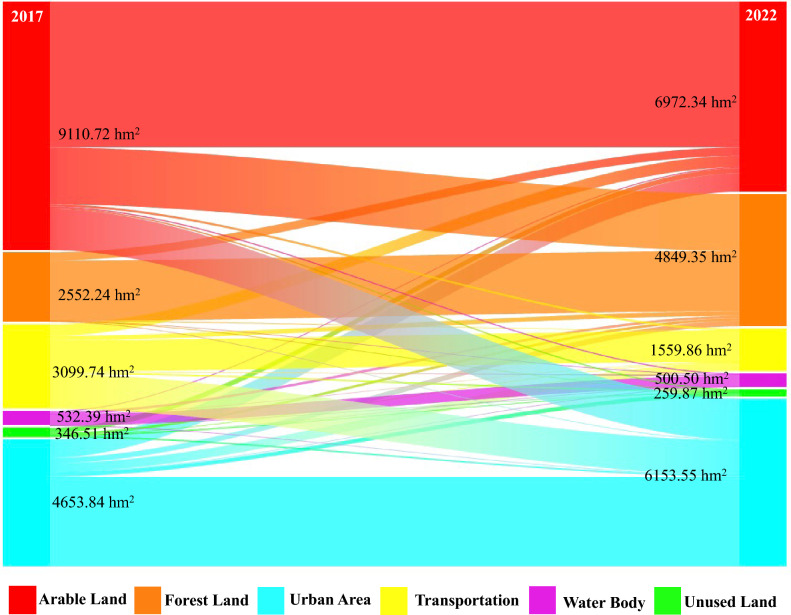
The Sankey diagram illustrating the Land Use Area Transfer Matrix from 2017 to 2022 (units: hm^2^). Generated in the Origin 2023 (https://www.originlab.com).

The land use transfer rules encompass two critical components: the land use transfer elasticity coefficient and the land use transfer matrix. The transfer elasticity coefficient, which ranges from 0 to 1, reflects the stability of a particular land use type. Smaller values indicate a higher likelihood of that land use type being converted to other types. In this study, we determined the transfer elasticity coefficients for Arable Land, Forest Land, Urban Area, Transportation, Water Body, Unused Land in Mianyang Science and Technology City New Area across three simulation scenarios, drawing from relevant literature^42,43^ and iterative parameter adjustments (Table 4). When combined with the 2017–2022 land use area conversion matrix for Mianyang Science and Technology City New Area, we generated a map (Fig. 6). The results highlight a significant increase in Urban Area, primarily driven by the conversion of Arable Land and Transportation. The Forest Land nearly doubles, mainly due to Arable Land conversion, while the Water Body and Unused Land remain largely unchanged, aligning with the current development trend. These parameters are subsequently integrated with the 2017–2022 land use area leveling results and the land use transfer matrix.

**Table 4:** The transfer elasticity coefficients assigned to each land use type in this study.

Land use scenario	Arable land	Forest land	Urban area	Transportation	Water body	Unused land
Baseline	0.7	0.6	0.9	0.9	0.9	0.2
Agricultural development	0.9	0.5	0.7	0.7	0.7	0.2
Construction development	0.5	0.5	0.9	0.9	0.7	0.2

These values range from 0 to 1, with smaller values indicating a higher likelihood of transfer to other land use types.

(2) Land use type requirements

Determining land use type requirements necessitates a method independent of the CLUE-S model. In this study, we determined the area requirements for different land use types in the study area for the year 2030 under various simulation scenarios. This was achieved using a linear interpolation method, considering different land use scenarios.

#### Accuracy assessment

To evaluate how closely the CLUE-S model simulations match real-world data, we use the quantity disagreement and allocation disagreement, which offers a comprehensive measure of agreement between the model's results and actual observations. Because of the two simple measures of quantity disagreement and allocation disagreement are much more useful to summarize a cross-tabulation matrix than the various Kappa indices for the applications that we have seen. These measurements can be computed easily by entering the cross-tabulation matrix into a spreadsheet available free of charge at^44^.

Quantity Disagreement and Allocation Disagreement are metrics used to assess the consistency between different data sources or algorithms. Quantity Disagreement measures variance in quantity, while Allocation Disagreement evaluates spatial disparities in land cover classification. Both metrics range from 0 to 1, with lower values indicating greater consistency and 0 representing perfect agreement, while 1 signifies complete disagreement or incongruity.

## Results and analysis

### Precision analysis of CLUE-S model simulation results

**Figure 7:**
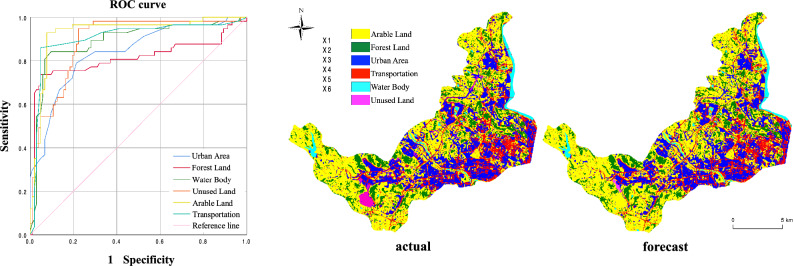
The ROC curve plot (left, where a curve closer to the upper-left corner indicates higher ROC values and thus higher model prediction accuracy) and the comparison plot of actual and forecast land use classification for the year 2022 (right). Generated in the IBM SPSS Statistics 26 (https://www.ibm.com/spss) and ArcGIS 10.6 software (www.esri.com).

The objective of this study was to evaluate the high-accuracy predictive capabilities of the CLUE-S model in a small-scale context. Our goal was to verify the model's feasibility and the related parameter settings. To achieve this, we employed the 2017 land use data to predict the 2022 land use data and compared these predictions with actual values to avoid drawing conclusions based on previous concerns regarding model accuracy. The data were subjected to logistic regression analysis using SPSS, and ROC (Related Operating Characteristic) curves (Fig. 7 left) were generated to evaluate the model's similarity to actual data. The Auto-Logistic regression equation test results indicated that the model successfully simulated the probability distribution of each land use class within the Mianyang Science and Technology City's new area. ROC values for Arable Land, Forest Land, Urban Area, Transportation, Water Body, Unused Land types all exceeded 0.7, meeting the regression requirements of the model. This affirmed that the selected factors could effectively simulate land use changes within the study area (Table 5). Based on the 2017 land use data for Mianyang Science and Technology City New Area, we simulated the land use layout for 2022. This simulation incorporated relevant data such as restricted areas, land transfer rules, land use demand, and Auto-Logistic model regression results into the CLUE-S model (Fig. 7). There are 150 well-distributed random samples used as ground truth data to assess the accuracy of the classification. The indexes of overall accuracy, quantity disagreement and allocation disagreement were calculated by using section “The CLUE-S model”. The quantity disagreement and allocation disagreement were 1.7% and 2.2%, respectively, indicating a high degree of agreement between the model and actual data. The overall simulation accuracy was an impressive 98.19%. Notably, the only discrepancy observed was in the southwestern part of the study area, where the prediction of unutilized land did not align with the actual situation. This discrepancy is attributed to government development policies and is considered an unavoidable error. Apart from this, the simulation results consistently matched the current situation. This result highlights the model's effectiveness in simulating land use changes and its suitability for predicting future developments within the new area of Mianyang Science and Technology City.

**Table 5:** The Auto-Logistic model regression analysis and display of β values for all influencing factors, resulting in ROC values for each land use type.

Driving factor	β coefficient					
	Arable land	Forest land	Urban area	Transportation	Water body	Unused land
River*	− 0.434	0.663	− 0.343	− 1.365	0.172	1.943
Transportation*	− 0.360	0.560	0.554	− 1.311	2.123	0.293
Township point*	− 0.608	5.049	1.131	− 0.936	− 1.148	− 1.751
Natural village*	− 1.839	− 1.518	− 2.238	1.817	− 2.203	− 1.116
Slope	− 2.026	− 0.421	− 1.249	− 5.503	0.996	1.668
Aspect of slope	0.863	− 0.499	0.365	1.511	0.483	− 4.369
Population density	0.654	− 0.244	0.520	6.812	− 1.841	− 0.770
Auto-value	10.751	20.128	10.978	13.032	18.576	14.152
Constant	− 4.168	0.296	− 6.621	− 1.431	− 1.691	− 3.552
ROC value	0.984	0.741	0.801	0.910	0.883	0.829

The ROC values range from 0 to 1, with higher values indicating higher model accuracy.

*Euclidean distance.

### Analysis of land-use change characteristics and scenario simulation

#### Land use scenario analysis for 2030

In this section, we analyze the land use scenarios for 2030, considering the natural resource status, economic and social development requirements, and development strategies of Mianyang Science and Technology City New Area. Three scenarios were constructed, each with distinct characteristics:

1. Baseline scenario

The baseline scenario represents the natural evolution of the study area, maintaining consistent development policies. In this scenario, Mianyang Science and Technology City New Area continues the land use policies of 2017–2022, with land demand changing at a constant rate based on the average rate of change for each category during this period. Specifically, Arable Land increases by 7.88% (from 6110.72 to 6586.18 hm^2^), Forest Land increases by 11.66% (from 3552.20 to 3966.56 hm^2^), Urban Area decreases by 3.51% (from 6653.84 to 6420.27 hm^2^), Transportation increases by 31.06% (from 1800.74 to 2360.10 hm^2^), Water Bodydecreases by 3.50% (from 632.39 to 610.24 hm^2^), and Unused Land decreases by 73.85% (from 1346.51 to 352.05 hm^2^).

(2) Agricultural development scenario

The Agricultural Development Scenario represents the spatial expansion of agricultural production. In this scenario, Mianyang Science and Technology City New Area ensures an adequate supply of agricultural land. Existing agricultural land remains stable, and efforts prioritize meeting the demand for land for agricultural development. Urban Area, Unused Land, and Forest Land are converted into Arable Land through initiatives like rural settlement reclamation, Unused Land development, and Forest Land restructuring. By 2030, the Arable Land in Mianyang Science and Technology City New Area increases by 29.70% compared to 2022, reaching 7925.68 hm^2^. Forest Land decreases by 13.94% (from 3552.20 to 3056.82 hm^2^), Urban Area decreases by 1.11% (from 6653.84 to 6579.51 hm^2^), Transportation increases by 1.57% (from 1800.74 to 1772.45 hm^2^), Water Body increases by 1.32% (from 632.39 to 640.78 hm^2^), and Unused Land decreases by 77.72% (from 1346.51 to 299.89 hm^2^).

(3) Construction development scenario

The Construction Development Scenario represents the spatial expansion of construction and development, primarily driven by the secondary and tertiary industries. In this scenario, Mianyang Science and Technology City New Area actively promotes the conversion of Arable Land, Forest Land and Unused Land (except permanent basic Arable Land) into Urban Area. All expansion potentials of rural settlements and Urban Area are realized, with a priority on meeting the demand for land for economic development. By 2030, almost all land categories are converted to Urban Area, with the area of other Unused Land reduced by 99.48%. Specifically, Urban Area increases by 22.15% (from 6653.84 to 8128.02 hm^2^), Arable Land increases by 1.72% (from 6110.72 to 6216.37 hm^2^), Forest Land increases by 8.10% (from 3552.20 to 3840.00 hm^2^), Transportation increases by 8.87% (from 1800.74 to 1960.60 hm^2^), Water Body increases by 4.22% (from 632.39 to 659.08 hm^2^).

#### Simulation analysis of spatial distribution of land use in 2030

**Figure 8:**
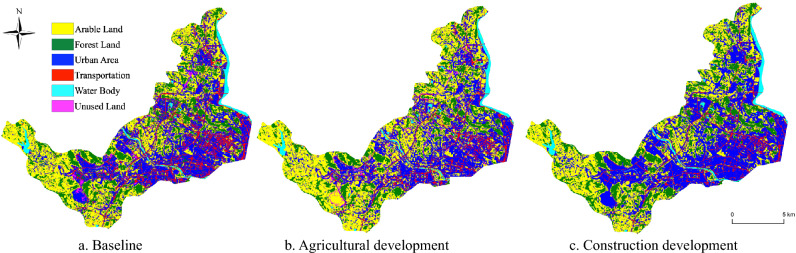
Land use distribution maps under three prediction scenarios for the year 2030. Generated in the ArcGIS 10.6 software (www.esri.com).

To forecast the spatial distribution of land use in Mianyang Science and Technology City New Area for the year 2030, we relied on the area specifications delineated in the three scenarios (Table 6). These scenarios encompass the Baseline Scenario, Agricultural Development Scenario, and Construction Development Scenario. The CLUE-S model was utilized for this simulation, commencing from the land use data in 2022 as the initial state. The simulation process incorporated the elasticity coefficients of land use transfer and the land use transfer matrices specific to each scenario. This enabled us to produce land use type maps for Mianyang Science and Technology City New Area under each of the three scenarios (Fig. 8a–c). This simulation yields valuable insights into the prospective spatial distribution of land use in the new area of Mianyang Science and Technology City in the year 2030 under diverse development strategies.

**Table 6:** The area of various land use types under three different scenarios for the year 2030 (units: hm^2^).

Land class	2030 scenario simulation		
	Baseline	Agricultural development	Construction development
Arable land	6586.18	7925.68	6216.37
Forest land	3966.56	3056.82	3840
Urban area	6420.27	6579.51	8128.02
Transportation	2363.7	1772.45	1460.6
Water body	610.26	640.78	659.08
Unused land	352.05	299.89	6.97

1. Baseline scenario (Fig. 8a)

In the baseline scenario, the new area of Mianyang Science and Technology City undergoes significant changes. The most notable transformation is observed in the unutilized land at the intersection of Mujia and Hebian townships in the southwest, which experiences substantial alterations. These changes are driven by Mianyang's development policy, which emphasizes growth in the northeast and southwest regions. This area experiences a substantial alteration, while other land types undergo relatively normal changes. Arable Land and Forest Land expand to varying degrees, with Transportation seeing a more pronounced increase.

(2) Agricultural development scenario (Fig. 8b)

Under the agricultural development scenario, agricultural land in the new area of Mianyang Science and Technology City remains stable and undergoes substantial expansion, which bolsters food security. However, this expansion places notable pressure on Forest Land and leads to a significant reduction in the area of Water Body. However, in this scenario, despite the concentration and continuous distribution of agricultural land in the southwestern region, Forest Land area shrinks notably due to the dual pressure of land and water resource demands. Additionally, the area of Water Body shows a significant reduction. This suggests that the agricultural development scenario may have a more pronounced impact on the ecological and water security of the new area of Mianyang Science and Technology City.

(3) Construction development scenario (Fig. 8c)

In the construction development scenario, there is a remarkable increase in the area of Urban Area, driven by a substantial conversion of Arable Land and Forest Land to fulfill economic development requirements. However, this expansion leads to the further fragmentation of agricultural and ecological land, posing potential threats to food security and ecological safety. This scenario witness’s significant expansion of construction land in the central and western regions, including urban cores, and a rapid increase in the area of rural settlements. Consequently, there is a further fragmentation of agricultural and ecological land, posing a serious threat to food security and ecological safety. This occurs despite the region's ability to meet its land requirements for economic development.

These scenario analyses shed light on the potential outcomes and implications of different development strategies for the new area of Mianyang Science and Technology City.

### Analysis of carbon emissions changes

**Figure 9:**
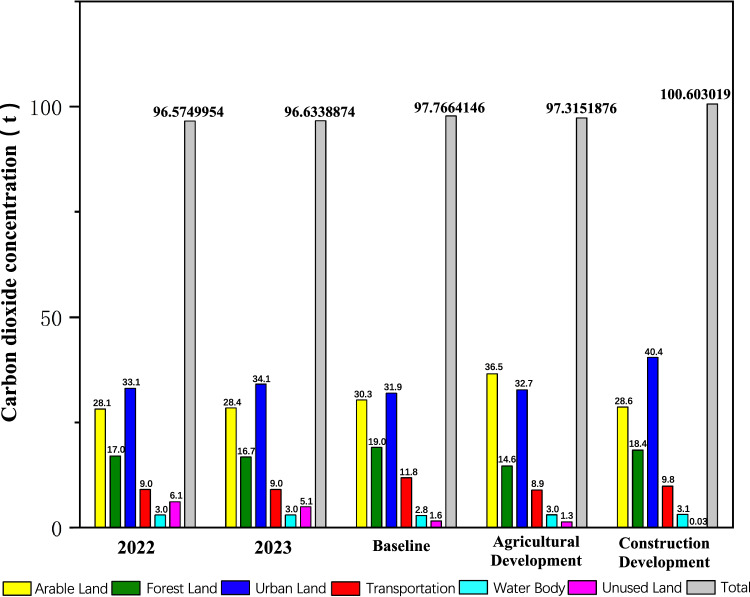
A bar chart comparing the data of CO_2_ concentration values and total emissions for various land use types in 2022 (actual measurement), 2023 (prediction and verification scenarios), and 2030 (three different prediction scenarios). Generated in the Origin 2023 (https://www.originlab.com).

Taking into account the CLUE-S simulation results and carbon emission data, we calculated the regional carbon emissions for Mianyang Science and Technology City New Zone in 2030 under various simulation scenarios and validation group data for 2023. (Table 7). Generate the conclusive carbon emission quantitative research overview (Fig. 9) based on the data presented in Table 7.

**Table 7:** Statistical data of CO_2_ concentration values and total emissions for various land use types in 2022 (actual measurement), 2023 (prediction and verification scenarios), and 2030 (three different prediction scenarios) (units: t).

Land class	2022	2023	2030		
			Baseline	Agricultural development	Construction development
Arable land	28.1712883	28.4718888	30.36322656	36.5385121	28.6583499
Forest land	17.048807	16.7863726	19.03753057	14.6712275	18.430105
Urban area	33.1347681	34.1273622	31.97163702	32.7646198	40.4758842
Transportation	9.07097626	9.07123149	11.88867414	8.92846934	9.87624868
Water body	3.0038442	3.00423424	2.89863199	3.04369659	3.13062135
Unused land	6.14531147	5.17279808	1.606714322	1.36866229	0.03181025
Total	96.5749954	96.6338874	97.7664146	97.3151876	100.603019

Based on the data presented in Table 7, we can draw the following conclusions: (1) The carbon emissions for Mianyang Science and Technology City New District in the year 2022 were recorded at 96.57 tons. (2) Projections for carbon emissions in Mianyang Science and Technology City New Area for the year 2030, considering different simulation scenarios, are as follows:

1. Baseline Scenario: 97.77 tons
2. Agricultural Development Scenario: 97.312 tons
3. Construction Development Scenario: 100.60 tons

Compared to the emissions in 2022, this represents an increase in carbon emissions of 1.233% under the Baseline Scenario, 0.766% under the Agricultural Development Scenario, and the highest increase of 4.170% under the Construction Development Scenario.

These empirical results elucidate that amidst the triad of modeled scenarios, carbon emissions attain their apogee within the precincts of the Construction Development Scenario. Such augmentation can be ascribed to the ascension in energy utilization and concomitant carbon emissions stemming from the vicissitudes of construction undertakings and urban sprawl. Conversely, the Agricultural Development Scenario exhibits lower carbon emissions, which may be attributed to improved management and efficiency in the agricultural production process. Carbon emissions under the Baseline Scenario fall between these two extremes.

Through the analysis of the 2023 validation group data, it is evident that there are minor fluctuations in data for various land categories. Due to changes in relative area, the impact of Unused Land and Forest Land on CO_2_ has slightly diminished. Overall, consonant with the overarching trajectory, the aggregate CO_2_ emissions persevere in their annual ascent antecedent to cresting at the carbon pinnacle in 2030, albeit at a conspicuously mitigated pace of expansion.

### Validation section

We did the whole process of chapter 3 from the beginning again after the above study, again using the CLUE-S model to import the data from 2017 to 2022, only this time the prediction was aimed at 2023, and through the method described in chapter 3, we inverted to get the predicted value of CO_2_ for the whole year of 2023 as 96.6338874 tones. Finally, after verifying the predicted value of CO_2_ in comparison with our actual measured value for the whole year of 2023: 96.62 tones, it was found that the prediction accuracy was more than 99.5%. Then we revalidated the accuracy of our model using the method outlined in Section “The CLUE-S model”. There are 150 well-distributed random samples used as ground truth data to assess the accuracy of the classification. When comparing the predicted and actual data for the year 2023. The quantity disagreement and allocation disagreement were 1.3% and 1.4%, respectively, indicating a high degree of agreement between the model and actual data. The overall simulation accuracy was an impressive 98.76%. This finding not only validates the feasibility of the entire study process, but also highlights its high degree of accuracy. Thus, we were able to confirm that a closed validation loop had been successfully formed for the entire study.

## Discussion and conclusions

### Discussion

In this investigation, we have employed the CLUE-S model, conjoined with empirically measured carbon dioxide concentration data, to undertake a comparative analysis of simulations of land use scenarios and carbon emissions at the county level. This endeavor serves as a scientific foundation for future county-level land use planning and the pursuit of carbon neutrality and peaking.

Through a thorough examination of land use and carbon emissions in the Mianyang Science and Technology City New Area in 2022, under various scenarios (Fig. 9), we can formulate the following integrated conclusions:

1. Impact of land use types on carbon emissions

It is apparent from these scenarios that land use types exert a substantial impact on regional carbon emissions. Forest Land emerges as a key determinant influencing carbon emissions owing to its robust capacity for carbon absorption, thereby assisting in the alleviation of carbon emissions. Conversely, roads, acting as a significant contributor to carbon emissions, exert a considerable influence on regional carbon emissions.

(2) Construction development scenario

In the construction and development scenario, there is a notable expansion in roads and urban areas, leading to a considerable rise in carbon emissions. This escalation can be attributed to increased carbon emissions arising from activities such as transportation and energy consumption during road construction and urbanization. However, it is imperative to note that the ecological resource advantage is considerably compromised under this scenario.

(3) Agricultural development scenario

In the agricultural development scenario, although the expansion of Arable Land and Forest Land may sequester some carbon, it could potentially impede regional economic growth due to the extensive conversion of land for construction purposes.

In summary, diverse land use types and development scenarios have significant ramifications on regional carbon emissions. Effectively addressing carbon emission reduction requires a nuanced approach that balances economic growth with ecological preservation. Striking this equilibrium is vital for minimizing carbon emissions while safeguarding precious ecological resources.

In this paper, we introduced the Auto-Logistic regression model to analyze the driving factors behind regional land use changes. This integration significantly improved the accuracy of CLUE-S model predictions, achieving an impressive verification accuracy of 98.19%. Furthermore, we systematically categorized and summarized land use data based on the relevant IPCC guidelines, enhancing the comprehensiveness and accuracy of our data sources. Consequently, our study results are highly practical and carry significant implications for county-level land use planning and the formulation of carbon emission reduction policies.

Challenges and considerations were addressed through comparisons with previous studies: (1) Difficulty of Data Acquisition and Processing: Land use carbon emission studies demand copious observational data and field sampling, which can be intricate and time-consuming^45^. (2) Uncertainty and Complexity: Carbon emissions from land use are influenced by a multitude of factors, leading to varying degrees of uncertainty and complexity in different regions and under different land use types^46^. (3) Spatial and Temporal Scale Limitations: The dynamic nature of land use change and carbon emissions often restricts studies to specific spatial and temporal scales, which may not encompass all change scenarios^47^. In our approach, we utilized actual CO_2_ concentration data rather than relying solely on calculation methods. This methodological choice eliminates accidental errors linked to seasonal fluctuations, temperature, anthropogenic factors, and weather conditions. Our combination of remote sensing, CLUE-S modeling, and surface observation data allowed for the simulation and evaluation of land use change impacts on carbon emissions and carbon stock. This holistic model application enhances our understanding of the intricate relationship between land use changes, the carbon cycle, and climate change, providing valuable support for informed land management and carbon emission reduction strategies.

Due to various factors, including policy changes and socio-economic development, regional land use and carbon emission efficiency may undergo significant changes in the future. Consequently, the predictive results may deviate from reality to some extent. A study conducted by^48^ utilized the CLUE-S model to investigate the impact of cross-scale land use changes on global land allocation. This study pointed out that the CLUE-S model might compromise the accuracy of simulation results due to its limited consideration of cross-scale interactions. Another study by^49^ applied the CLUE-S model to analyze the effect of urban expansion on agricultural land use intensity in China. However, the model's oversimplified representation of the land use decision-making process could introduce uncertainty into the simulation outcomes. Furthermore,^50^ employed the CLUE-S model to simulate land use changes in the urban–rural interface areas of the Beijing–Tianjin–Hebei region in China. Nevertheless, the model's spatial resolution and data availability limitations may restrict the accuracy of the simulation results. In a study by^51^, a CLUE-S model in conjunction with a Markov model was used to simulate land use changes in the Cerrato region of Brazil. It's important to acknowledge that future changes in regional land use and carbon emission efficiency may be subject to significant shifts due to evolving policies and socio-economic developments. Predictive results may inherently contain errors when compared to real-world outcomes. Addressing these challenges is essential for refining the accuracy and practical utility of land use and carbon emissions modeling.

In conclusion, our study marks a substantial advancement in comprehending the intricate interplay between land use modifications and carbon emissions at the county level. To augment the applicability of our findings, future investigations should delve into supplementary driving forces, including socio-economic and policy influences, and contemplate the nuanced transformation processes of distinct land use categories to enhance the refinement of data analysis and application.

### Conclusion

In this investigation, we utilized Sentinel-2A remote sensing data covering the period from 2017 to 2022 for the Mianyang Science and Technology City New Area. We employed the CLUE-S model to simulate and validate land use change patterns for the year 2022. Subsequently, we formulated three distinct scenarios—the baseline scenario, agricultural development scenario, and construction development scenario—to simulate the land use arrangement in 2030 and evaluate its corresponding carbon emissions. Our analysis yields the subsequent key conclusions:

1. Our study demonstrates the robust simulation capabilities of the CLUE-S model concerning land use layout changes within the study area. The model achieved quantity disagreement and allocation disagreement were 1.7% and 2.2%, indicating a high level of agreement with observed data. The overall simulation accuracy reached an impressive 98.19%. These results affirm the suitability of the model and its pertinent parameters for predicting future land use layouts in Mianyang Science and Technology City New Area.
2. Under the three distinct simulation scenarios, land use layout in 2030 exhibits diverse characteristics:
  - The construction and development scenario demonstrates optimal land resource utilization for short-term development but is concurrently associated with heightened pollution concerns.
  - The baseline scenario portrays a more gradual and sustainable development trajectory suitable for medium- and long-term goals.
  - The agricultural development scenario succeeds in achieving lower surface CO_2_ concentrations in alignment with the objectives of “peak carbon” and “carbon neutrality.” However, it comes at the cost of substantial ecological and water resource degradation.
  In summary, each of these simulation scenarios carries its own set of development risks: the baseline scenario poses threats to ecological and food security in certain regions due to a deteriorating land-use structure; the agricultural development scenario prioritizes food security but at the expense of ecological and water resources; and the construction development scenario, while ensuring land availability for economic growth, faces significant challenges concerning food security and ecological safety.
3. Compared to the year 2022, the overall carbon emissions in the Mianyang Science and Technology City New Area are on the rise. Under the scenario of agricultural development, the carbon emissions show the smallest increase, at 0.766% (97.36 tons). In contrast, under the scenario of construction industry development, the carbon emissions exhibit the highest increase, reaching 4.170% (exceeding 100 tons).
4. The predictive accuracy of the validation group's CO_2_ concentration values can reach 99.5%.

Our study reveals crucial relationship into county-level land use patterns and carbon emissions across various simulation scenarios. These findings constitute a crucial scientific basis for future regional development efforts. Nonetheless, considering the potential challenges inherent in these scenarios, policymakers and planners should exercise careful consideration of diverse factors to realize the sustainable development goals. Subsequent research should delve further into the underlying mechanisms of impact across various scenarios, while integrating a broader range of driving factors to offer even more comprehensive and precise decision-making support.

### Supplementary Information

Supplementary Information.

## Data Availability

The datasets generated and analysed during the current study are not publicly available due [The research area may involve the sensitive military industrial area of China's nuclear research, and the continuous CO_2_ data measured by us can be used for this study, but it is not convenient for public display. Other basic data is provided for download on the website, the processing process is too cumbersome and most of the results are obtained using common processing methods.] but are available from the corresponding author on reasonable request.
