# Supplementary material for: County land use carbon emission and scenario prediction in Mianyang Science and Technology City New District, Sichuan Province, China
Source: Sci Rep. 2024 Apr 23;14:9310. doi: 10.1038/s41598-024-60036-3 (PMC11039458; doi:10.1038/s41598-024-60036-3)
Supplement: Supplementary file 1 — Supplementary Information. [file 41598_2024_60036_MOESM1_ESM.docx]

**Appendix:**

Extract CO2 value codes for each class unit area:

import numpy as np

import cv2

co2_avg = cv2.imread(r"D:\co2_avg.jpg")

mianyang_map = cv2.imread(r"D:\mianyang_map.jpg") #BGR

h, w = co2_avg.shape

colors_maps, colors_co2 = [], []

# Traverse the vector graph

for idx in range(h):

for jdx in range(w):

# Filter white (accelerated)

if mianyang_map[idx, jdx].all() == 255 and co2_avg[idx, jdx].all() == 255:

continue

# The color of the current pixel (format BGR)

color_mianyang = list(mianyang_map[idx, jdx])

color_co2 = list(co2_avg[idx, jdx])

if color_mianyang not in colors_maps:

colors_maps.append(color_mianyang)

if color_co2 not in colors_co2:

colors_co2.append(color_co2)

bgr_colors_map = np.array(colors_maps)

bgr_colors_co2 = np.array(colors_co2)

#-------Here is the segmentation, the above is the preliminary processing work----------#

#Get the content corresponding to each color in the image

bgr_colors_map = np.array([[0, 255, 255], [1, 128, 0], [0, 0, 254], [255, 255, 0], [196, 255, 1], [254, 0, 0]])

bgr_colors_co2 = np.array([[0, 167, 56], [1, 189, 96], [0, 209, 139], [1, 232, 193],

[0, 255, 255], [0, 190, 255], [0, 127, 255], [1, 64, 255], [0, 0, 254]]) #Co2 concentration from low to high

co2_avg_maps = np.zeros((h, w))

#Cropland woodland road water body other town all coordinates on the vector map

mask_gendi = cv2.inRange(mianyang_map, bgr_colors_map[0], bgr_colors_map[0])

mask_lindi = cv2.inRange(mianyang_map, bgr_colors_map[1], bgr_colors_map[1])

mask_daolu = cv2.inRange(mianyang_map, bgr_colors_map[2], bgr_colors_map[2])

mask_shuiti = cv2.inRange(mianyang_map, bgr_colors_map[3], bgr_colors_map[3])

mask_qita = cv2.inRange(mianyang_map, bgr_colors_map[4], bgr_colors_map[4])

mask_chengzhen = cv2.inRange(mianyang_map, bgr_colors_map[5], bgr_colors_map[5])

#The regional coordinates of the 9 Co2 concentration regions on the vector diagram

mask_co2_369 = cv2.inRange(co2_avg, bgr_colors_co2[0], bgr_colors_co2[0])

mask_co2_374 = cv2.inRange(co2_avg, bgr_colors_co2[0], bgr_colors_co2[0])

mask_co2_378 = cv2.inRange(co2_avg, bgr_colors_co2[0], bgr_colors_co2[0])

mask_co2_383 = cv2.inRange(co2_avg, bgr_colors_co2[0], bgr_colors_co2[0])

mask_co2_388 = cv2.inRange(co2_avg, bgr_colors_co2[0], bgr_colors_co2[0])

mask_co2_392 = cv2.inRange(co2_avg, bgr_colors_co2[0], bgr_colors_co2[0])

mask_co2_397 = cv2.inRange(co2_avg, bgr_colors_co2[0], bgr_colors_co2[0])

mask_co2_402 = cv2.inRange(co2_avg, bgr_colors_co2[0], bgr_colors_co2[0])

mask_co2_406 = cv2.inRange(co2_avg, bgr_colors_co2[0], bgr_colors_co2[0])

#Create an image of the same size as the vector diagram and assign each Co2 coordinate to the corresponding concentration

co2_avg_maps[mask_co2_369] = 369.3032769

co2_avg_maps[mask_co2_374] = 374.0106676

co2_avg_maps[mask_co2_378] = 378.7180583

co2_avg_maps[mask_co2_383] = 383.4254489

co2_avg_maps[mask_co2_388] = 388.1328396

co2_avg_maps[mask_co2_392] = 392.8402303

co2_avg_maps[mask_co2_397] = 397.5476210

co2_avg_maps[mask_co2_402] = 402.2550117

co2_avg_maps[mask_co2_406] = 406.9624023

#Average Co2 concentration in Arable Land

avg_co2_gendi = sum(co2_avg_maps[mask_gendi]) / len(mask_gendi[mask_gendi==True]) #Total area concentration number/total area area

#Average Co2 concentration in Forest Land

avg_co2_lindi = sum(co2_avg_maps[mask_lindi]) / len(mask_lindi[mask_lindi==True])

#Average Co2 concentration in Transportation

avg_co2_daolu = sum(co2_avg_maps[mask_daolu]) / len(mask_daolu[mask_daolu==True])

#Average Co2 concentration in Water Body

avg_co2_shuiti = sum(co2_avg_maps[mask_shuiti]) / len(mask_shuiti[mask_shuiti==True])

#Average Co2 concentration in Unused land

avg_co2_qita = sum(co2_avg_maps[mask_qita]) / len(mask_qita[mask_qita==True])

#Average Co2 concentration in Urban Area

avg_co2_chenzheng = sum(co2_avg_maps[mask_chengzhen]) / len(mask_chengzhen[mask_chengzhen==True])
